# Enhanced ranking of PknB Inhibitors using data fusion methods

**DOI:** 10.1186/1758-2946-5-2

**Published:** 2013-01-14

**Authors:** Abhik Seal, Perumal Yogeeswari, Dharmaranjan Sriram, OSDD Consortium, David J Wild

**Affiliations:** 1 School of Informatics and Computing, Indiana University Bloomington, Bloomington, IN, 47408, USA; 2 Computer-Aided Drug Design Laboratory, Department of Pharmacy Birla Institute of Technology, Hyderabad Campus, Shameerpet, Hyderbad, 500078, India; 3 Open Source Drug Discovery, Council of Scientific and Industrial Research, New Delhi, India

**Keywords:** PknB, Virtual screening, Data fusion, BEDROC, Reciprocal rank

## Abstract

**Background:**

Mycobacterium tuberculosis encodes 11 putative serine-threonine proteins Kinases (STPK) which regulates transcription, cell development and interaction with the host cells. From the 11 STPKs three kinases namely PknA, PknB and PknG have been related to the mycobacterial growth. From previous studies it has been observed that PknB is essential for mycobacterial growth and expressed during log phase of the growth and phosphorylates substrates involved in peptidoglycan biosynthesis. In recent years many high affinity inhibitors are reported for PknB. Previously implementation of data fusion has shown effective enrichment of active compounds in both structure and ligand based approaches .In this study we have used three types of data fusion ranking algorithms on the PknB dataset namely, sum rank, sum score and reciprocal rank. We have identified reciprocal rank algorithm is capable enough to select compounds earlier in a virtual screening process. We have also screened the Asinex database with reciprocal rank algorithm to identify possible inhibitors for PknB.

**Results:**

In our work we have used both structure-based and ligand-based approaches for virtual screening, and have combined their results using a variety of data fusion methods. We found that data fusion increases the chance of actives being ranked highly. Specifically, we found that the ranking of Pharmacophore search, ROCS and Glide XP fused with a reciprocal ranking algorithm not only outperforms structure and ligand based approaches but also capable of ranking actives better than the other two data fusion methods using the BEDROC, robust initial enhancement (RIE) and AUC metrics. These fused results were used to identify 45 candidate compounds for further experimental validation.

**Conclusion:**

We show that very different structure and ligand based methods for predicting drug-target interactions can be combined effectively using data fusion, outperforming any single method in ranking of actives. Such fused results show promise for a coherent selection of candidates for biological screening.

## Background

Most people affected by tuberculosis contain both active infection and latent infection. The mechanism of Mycobacterium tuberculosis shift between the latent and active state is not clearly understood, but one of the essential components of these kind of systems is the regulation of cell wall synthesis and cell division in response to stimuli from the host via signal transduction. One of the chief mechanisms by which extracellular signals are translated to intracellular responses is via protein phosphorylation. In bacteria Protein phosphorylation is carried out by specific protein kinases in a two component system. In eukaryotes protein phosphatases and protein kinases plays a key role behind of host and pathogen signal transduction pathways. Mycobacterium tuberculosis having 11 serine-threonine proteins Kinases (STPKs) and it has been found that Ser/Thr kinases are an attractive target for drug discovery [1,2]. The 3D structures of PknB, PknE and PknG are required for mycobacterial growth [3] and are being deposited in PDB (http://htt://www.rcsb.org) which resemble the human kinases with conserved motif and a striking similarity of ATP bound kinase domain with the activated eukaryotic Ser/thr Kinases [4]. PknB is a receptor like protein transmembrane protein with an extracellular signal sensor domain (PASTA) and an intracellular kinase domain and it shares high sequence similarity with eukaryotic STPKs [4,5]. PknB is a very important functional protein kinase which can phosphorylate by itself. It has been shown that the expression of PknB is constitutive and is present under both in vitro and in vivo conditions. Previously MtrA was response regulator for Mycobacterium tuberculosis and found to be essential for growth [5].Further it was also observed Fernandez [6] that knock-outs and overexpression of PknB affects the cell morphology which supports involvement of with cell division and shape which shows that PknB is the right molecular target for designing inhibitors. There is a need for the design of new inhibitors because current drugs, whilst effective *in vitro* have not shown good *in vivo* activity. Our hypothesis is that this is because the compounds are not targeting PknB in cells. Though the sequence identity is less than 27% and the PknB structure shows a very low RMSD of 1.36 Å and 1.72 Å with eukaryotic kinases [7-9], the overall catalytic domain is similar to the eukaryotic protein kinase consisting the N terminal subdomain including a β-sheet and a long α-helix and the C terminal lobe consists of α-helices [10]. In this work we have used ligand and structure based approaches to screen large set of inhibitors. Previously many high affinity inhibitors have been reported for PknB [11-15]: we used 62 inhibitors listed in Additional file 1 for our work.

Virtual screening (VS) using structure and ligand based approaches is widely used in drug discovery [16]. Structure-based screening involves using information about a protein target, usually through molecular docking. It requires a protein structure to be known, but known active ligands are not required. Ligand-based screening only uses information from active ligands, but does not require a protein target structure. Both structure and ligand based approaches can be applied parallel to VS, but often these approaches are applied in a stepwise filtering approach [17]. The most commonly applied VS methods are molecular docking, pharmacophore identification and ligand similarity (including shape based), along with a variety of machine learning methods that “learn” to differentiate actives from inactives based on known data [18,19]. Simple similarity searching with known ligands can also be effective [20,21].

The most important challenge in VS is to create accurate scoring function that can distinguish between novel bioactive and inactive molecule. In case of docking the three classes of scoring is highlighted forcefield based scoring, empirical scoring and knowledge based scoring [22]. The three classes include various types of scoring algorithms are used for molecular docking, historically, scoring does not correlate well with binding activity, although Consensus scoring, which takes a weighted average of several methods, can result in improvements [23,24]. However, these consensus scores are only concerned with variations of a structure-based approach and their limitations have been documented [25]. Data Fusion has been shown to be effective in integrating data from different sources [26-28] for example Willet etal used 2D similarity searching using different similarity measures using SUM function, although there are few results reported using structure and ligand based approaches along with data fusion [29,30].

In this study we have applied multiple ligand and structure based methods to the PknB problem and then combined these results using data fusion. Performance was evaluated with a widely used benchmark dataset from Schrodinger (http://www.schrodinger.com/glidedecoyset), which has been used in other VS [31,32]. This set is a set of decoys that have similar properties to the active compounds but are topologically dissimilar. We also evaluated ranking of actives using a variety of well-established methods including Enrichment factor (EF) [31], RIE [33] and BEDROC [34,35]. We used the EF,RIE and BEDROC in the each of the VS protocols and in the data fusion algorithms such as sum score, sum rank and reciprocal rank and found that reciprocal rank outperformed all of the VS protocols such as pharmacophore search, shape screening and docking and as well as related to other algorithms. The next best algorithm which performed well in data fusion was sum score rank which outperformed the structure and ligand based approaches.

The aim of the study was to utilize several VS protocols and then fuse the results for evaluation the results and identify best fusion algorithm among the 3D structure based, ligand based methods and fusion algorithms, and to show how these results could be used to select compounds for follow-up testing.

## Methods

### Dataset preparation

**Figure 1:**
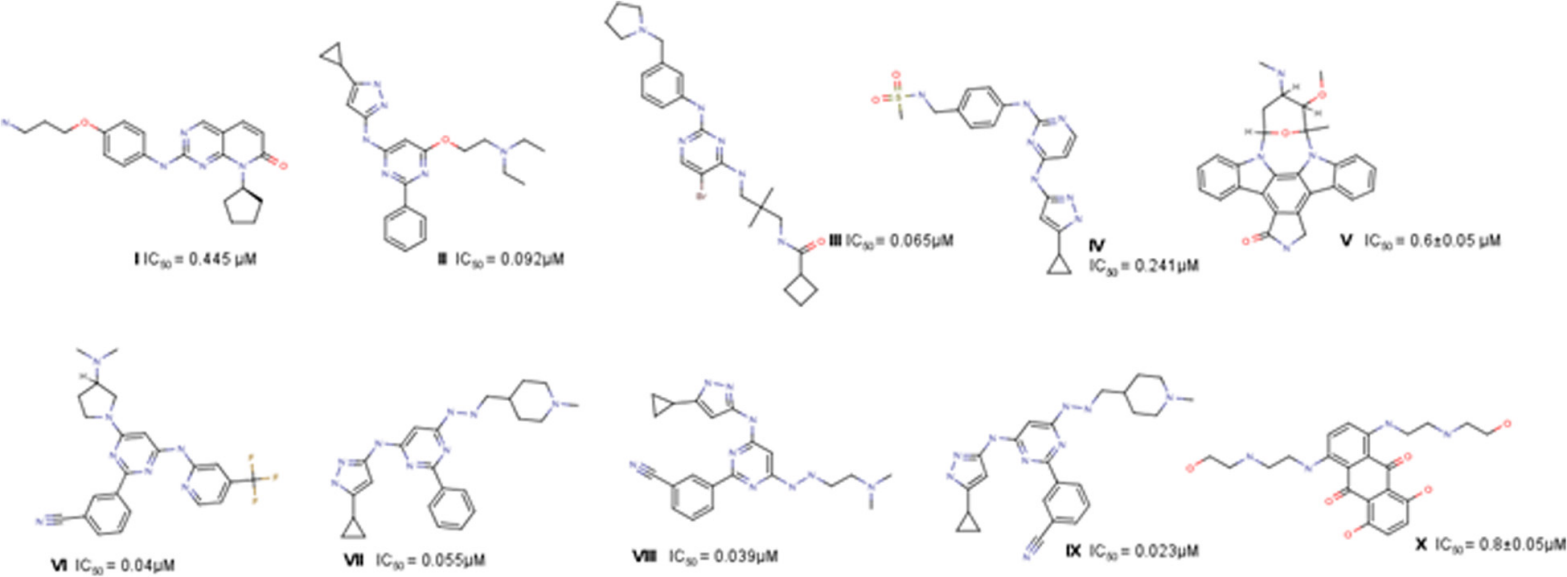
Some of the PknB inhibitors stsructures with IC_50_ values.

For PknB many inhibitors were earlier reported like aminopyrimidine analogues and aminopyrazoles [11-15]. The reported lists of 62 inhibitors were drawn in chemaxon’s Marvin sketch application [36]. Some of structures of active compounds are given in Figure 1. The decoy set of molecular weight 400Da was downloaded from the Schrodinger’s website. For screening freely available molecular database of Asinex was used in the study which consisted of 3, 93,000 molecules. All the structures were prepared using LigPrep (LigPrep v2.2, Schrodinger LLC, New York, NY) with Epik (Epik v1.6, Schrodinger, LLC, New York, NY) to expand protonation and tautomeric states at 7.0 ± 2.0 pH units.

Conformational sampling was performed on all database molecules using the ConfGen search algorithm [37]. Confgen with OPLS 2005 forcefield was applied for generation of conformers with duplicate poses eliminate if the RMSD is less than 1.0 Å. A distance dependent dielectric constant of 4 and maximum relative energy difference of 10 kcal/mol is applied as suggested by salam etal [38]. For validation of the structure and ligand based approaches we used a randomly selected set of 35 active compounds from the dataset of 62 active compounds and 1000 decoy compounds making a total of 1035 compounds as the database given in Additional file 2.

### Protein preparation

For protein preparation PknB inhibitor Mitoxantrone (Mtz) bound crystal structure PDBID: 2FUM was prepared using the protein preparation wizard. Bond orders and formal charges were added for hetero groups, and hydrogens were added to all atoms in the system. Water molecules were removed. A brief relaxation was performed using an all-atom constrained minimization carried out with the Impact Refinement module (Impref) (Impact v5.0, Schrodinger, LLC, New York, NY) using the OPLS-2005 force field to alleviate steric clashes that may exist in the original PDB structures. The minimization was terminated when the energy converged or the rmsd reached a maximum cutoff of 0.30 Å.

### Virtual screening methods

Three methods of VS were applied in the current study: Docking using Glide [32,37], e-pharmacophore search using phase [39] and 3D shape similarity search using vROCS [40]. Our aim was to investigate whether data fusion methods can search and rank actives from a database.

For docking, the extra precision (XP) mode was used both for the actives and decoy sets and all settings were left as default except for adding the Epik states penalties to the docking score. Glide energy grid was generated using 2FUM structure. It was found that the glide grid for the ligand was smaller using the default settings so it was extended to 12Å to cover all the ligands and the active site grid covered whole of the active site.

**Figure 2:**
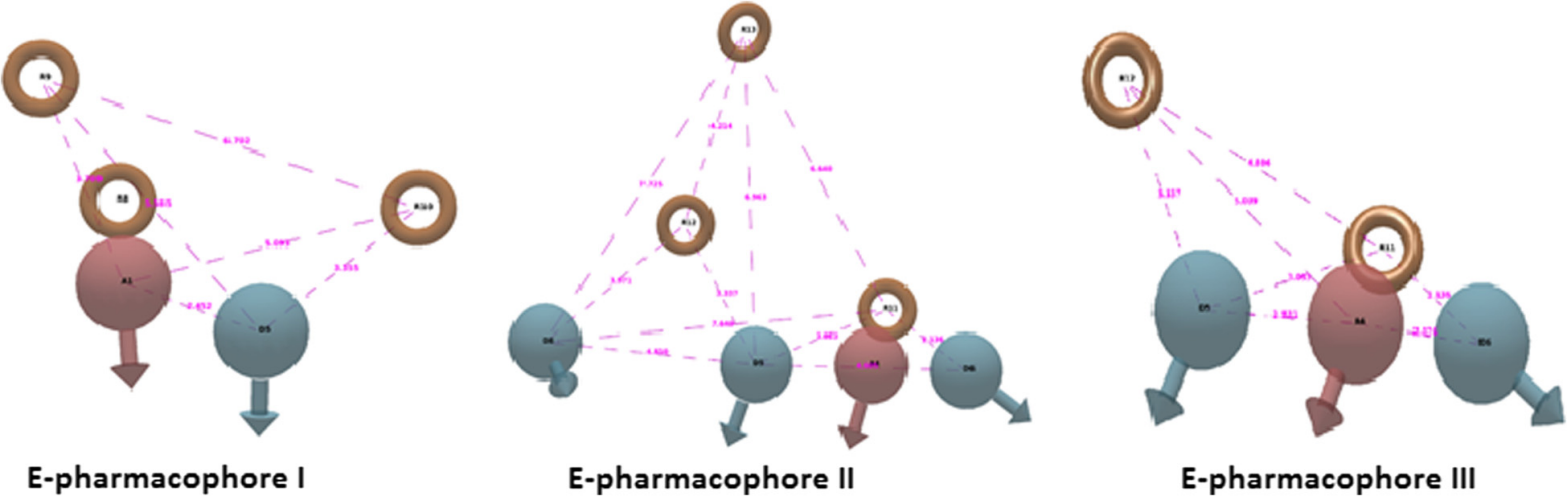
Shows the different pharmacohores developed by Phase E-pharmacophore.

To generate the pharmacophoric features we used the energetic pharmacophore as developed by Salam et al. It is a very useful method unlike ligand based pharmacophore which requires a set of structures and also skill in defining proper training and test sets. The e-pharmacophore can be developed with a single active compound. The 62 active compounds were docked into the crystalline ligand structure using the Glide XP refine docking algorithm. The Glide XP descriptors generated were used in building the e-pharmacophore from the Maestro Scripts menu option with default excluded volume option 0.5 pharmacophoric sites were automatically generated with Phase using the default set of six chemical features: hydrogen bond acceptor (A), hydrogen bond donor (D), hydrophobic (H), negative ionizable (N), positive ionizable (P), and aromatic ring (R). We generated 4 different e-pharmacophores for 2FUM given as Figure 2 and from that we have evaluated the best one using the statistical metrics discussed in results and discussion section. Phase findmatches was used to generate conformers of the compounds on the fly on screening the active and decoy sets. Partial matches down to three features were used to get the fitness measures of the active and decoy sets of 1035 compounds. For data fusion one important criteria we used was that we required all the compounds fitness measures and we screened the dataset with minimum two features which lead us to get the scores of compounds which was not listed in three feature scoring.

Using a python script we extracted the remaining hits and ranked according to their descending fitness values. For vROCS screening compounds containing it relevant conformers were generated using OMEGA [41] with a maximum of 1000 conformers for each molecule and using the parameters mentioned in Bostrom et al. [42]. The query for the ROCS was the Glide XP docked structure poses of the active compound **VIII**. We selected this compound because we used this compound’s e-pharmacophore in findmatches. The explanation can be found in the Results and Discussion section.

### Data fusion

Three different data fusion algorithms were used to generate data fusion ranks, namely, sum score, sum rank and reciprocal rank. None of these methods required any training set which was mandatory in a VS method. Earlier studies have reported some other similar methods of fusion [27,43-46]. In Sum score the relative score of each compound is calculated by dividing by the highest score attained by any compound in a screening method. The scores were calculated for each VS system and were then summed to give a new fused score. Sum rank added the ranks together retrieved from each VS method and assigned a new rank based on the combined rank value. The minimum rank value receives the best rank and the maximum rank value receives the worst rank [31]. The reciprocal rank [46-48] or the rank position method merged the results based on the rank positions. The rank positions were summed using the inverse of the rankings given by equation below.

(1) $$r\left(C_{i}\right)=\frac{1}{\sum_{j}1/\mathrm{pos}\left(C_{\mathrm{ij}}\right)}$$

where *C*_*i*_ is the rank of the compound i and j is the system index or the VS protocol used.

### Assessment of virtual screening methods

There are many metrics for evaluation of a predictive model such as precision ,recall, accuracy, FP measure. But all of them were not suitable for early recognition of true positives in a VS experiment. Each of the measures mentioned was used for special purpose for example in a classification problem [18,19]. To determine performance of the 3D methods used in the study, some important measures were considered i.e., yield of active compounds, percentage actives and Goodness of a Hit list (GH scoring) were considered. Percentage yield of actives was the ratio of actives found in the hit list to the total number of compounds in the hit list i.e. given by equation 2.

(2) $$\%\;\mathrm{yield}\;\mathrm{of}\;\mathrm{actives}\left(\mathrm{Ya}\right)=\frac{\mathrm{TP}}{\mathrm{TP}+\mathrm{FP}}\;\times \;100$$

Percent actives is the ratio of actives to the total number of actives (true postives) in the dataset given by equation 3.

(3) $$\%\;\mathrm{Actives}=\frac{\mathrm{TP}}{A}\times \;100$$

The Güner-Henry score i.e. Goodness of hit (GH score) [49-51] method consist of computing the sensitivity (recall) eq 4 and specificity (precision) eq 5 and enrichment factor. The GH score ranges from 0 and 1 where a value of 1 signifies an ideal model. Eq 6 gives the representation of GH score.

(4) $$\mathrm{Sensitivity}=\frac{\mathrm{TP}}{\mathrm{TP}+\mathrm{FN}}$$

(5) $$\mathrm{Specificity}=\frac{\mathrm{TN}}{\mathrm{TN}+\mathrm{FP}}$$

(6) $$\;\mathrm{GH}=\left(\frac{3}{4}.\mathrm{Ya}+\frac{1}{4}\;.\mathrm{Se}\right)\mathrm{Sp}$$

TP is the number of true positives returned after screening the database, TN, is the number of true negatives, FP, is the number of false positives ,FN, is the number of false negatives and A , is the total number of actives in the database.

To determine the top ranked compounds in a VS process one of the metrics used is the enrichment factor (EF) which is defined as the ratio of the probabilities of searching an active compound in top X% of the data set [52] given in eq 7.

(7) EnrichmentFactorEF=YaAD

Where D is the total number of database compounds.

Despite the early recognition problem the EF has some problems ignoring complete ranking of the whole dataset molecules [37,53].A method i.e. superior to random selection of compounds has EF > 1. To address the problem of EF as discussed in various reports [38,54] Sheridan et al. developed an exponential weighted scoring scheme RIE which gives heavier weight in “early recognized” hits [34,35].

(8) $$\mathrm{RIE}=\frac{\frac{1}{n}\sum_{i=1}^{n}e^{-\alpha {х}_{i}}}{\frac{1}{N}\left(\frac{1-e^{-\alpha }}{e^{\frac{\alpha }{N}-1}}\right)}$$

Where $X_{i}=\frac{r_{i}}{N}$ is the relative rank of the i^th^ active compound and α is the tunning parameter. Changing the parameter α, one can control the early ranking of hits. BEDROC values ranges in between [0,1] and can be defined as the probability that an active is ranked before a randomly selected compound was exponentially distributed with parameter α. BEDROC and RIE have a linear relationship [55].

We calculated the BEDROC value for three VS methods at α=20.At α=20 implies that 80% of the the final BEDROC score is based on the first 8% of the ranked data set.

### Virtual screening and data fusion of database hits

E-pharamcophore, vROCS, and glide SP docking were performed on Asinex datasets in a step by step process.To select the best scoring molecules from the database of Asinex we first screened the database using the e-pharmacophore matching at least 4 features out of five present in the Pharmacophore model. A distance matching with tolerance of 2 Å was given during pharmacophore mapping of the database along with a minimum of 4 sites to match the database entries. Also Excluded volumes were included in search generated from e-Pharmacophore. Phase find matches retrieved 5000 molecules from the database screen. For vROCS screening we used the 5000 molecules as the primary dataset and screened the dataset using the Glide XP docked pose of compound **VIII**. We also carried out Glide SP docking with the 5000 hits retrieved from phase find matches search. Docking was carried using the prepared protein 2FUM; the ligands were docked into the ATP binding site of the protein.

All the 5000 hits were ranked accordingly. For e-pharmacophore shortlisted hits were ranked in descending order, i.e. the highest fitness compound was given a best rank. ROCS automatically ranked compounds, based on the Tanimoto combo score. Flexible Glide SP docking was carried out and the ranking was done based on the docking energy. Then based on the scores and rankings sum score method was applied for ranking the hits. The sum score was selected since it produced the best results than the other fusion methods. After ranking and screening, the top 500 compounds (~10%) of the dataset was used for further evaluation. 500 compounds were further docked using the Flexible Glide XP docking. The binding poses were visually inspected for 500 molecules. The poses were compared with the pharmacophore alignment and the molecules which align in the same pattern were considered. The molecules with a maximum of at least 2 H-bonds with the hinge region at the ATP binding site were considered along with any one of the rings.

## Results and discussion

### E-pharmacophore generation

**Figure 3:**
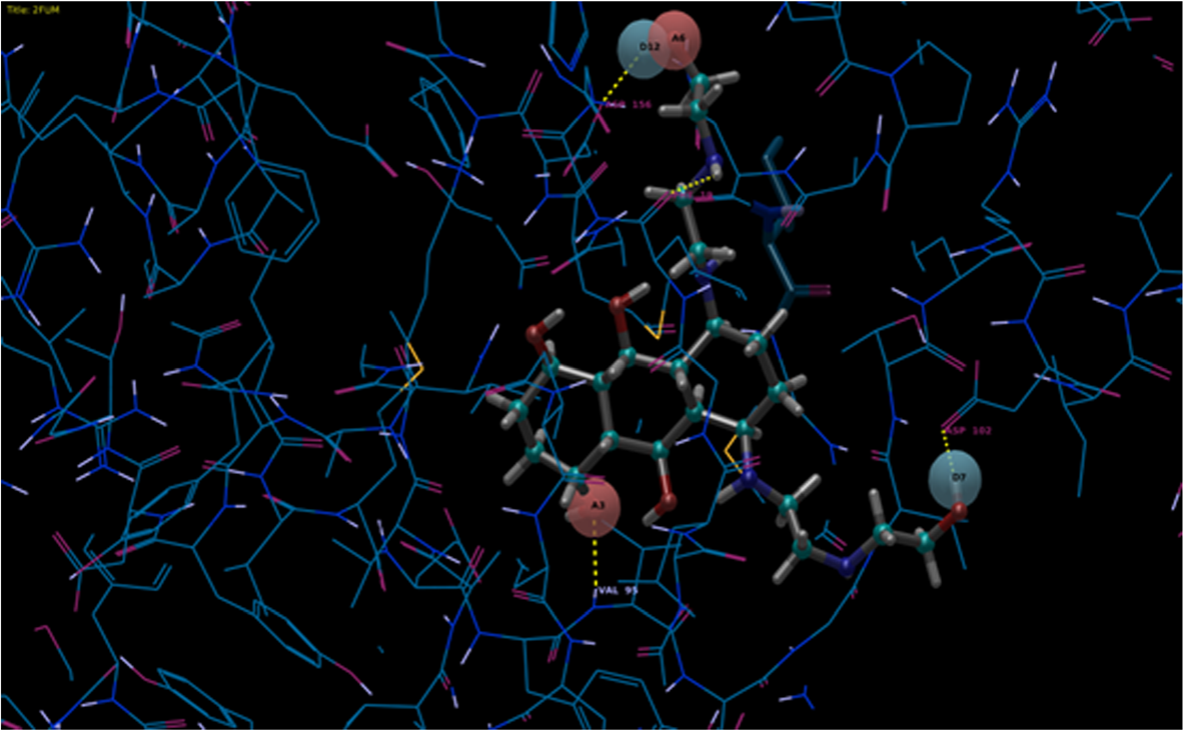
The picture represents the E-pharmacophore generated for Mitoxantrone.

Four e-pharmacophore models were generated using the six default features for Mitoxantrone (Mtz) and along with the top 2 docked compounds at the binding site. Though the methodology of e-Pharmacophore was based on the concept of structure based pharmacophore, the favorable pharmacophore sites generated from crystallized compound structure of Mtz were not able to retrieve much active compounds with high fitness scores. Figure 3 gives the view of e-pharmacophore of Mtz. Another most important aspect to consider was that kinases have specific type of pharmacophores as described by Zuccotto et al. [56] and e-pharmacophore of Mtz did not seem to correspond to any class of kinase pharmacophore. Kinase groups of drugs are mostly being classified into three types of pharmacophores type I, type II and type I½. Drugs such as sunitinb, erlotinib and dasatinib fall into the class I type of Inhibitors. It is found that type I inhibitors are ATP competitive and bind at the ATP site hinge region using one hydrogen bond acceptor, two hydrogen bond donors and a hydrophobic moiety [56]. We decided to dock the active set of 62 compounds to ATP binding site using Glide XP. After XP docking Compound **I** gave the best score of −10.766. Using this compound we generated the **epharmacohore I** with one acceptor, one donor and three rings as given in Figure 2. The pharmacophore almost resembled the type I of inhibitor except without one extra donor. The e-pharmacophores gave a score of −2.14 to the donor site D5 to Val95,-2.10 to the acceptor site A1 also binding to Val95.The other three were ring aromatics which are required for the hydrophobic interaction within the pocket.R10 showed a score of −1.05,R9 with −0.94 and R8 with −0.89. We also found another compound **VIII** with low inhibitory concentration (IC_50_) of 0.039μM on the second position of the docked poses. We used compound **VIII** to generate another set of **epharmacophore II** it which is shown in Figure 2.One Acceptor A4 of score −2.08 making interaction with Val95.Three donors namely D5 having score of −1.69 and other D6 and D8 having a score of −0.58 each. Three aromatic rings, R11 having a score of −0.65 and others R12 and R13 having a score of −0.67 each. Since seven sites were considered too many for a pharmacophore we removed R13 and D8 based on the must match option for this positions. For must match we used these two positions R13 and D8 along with any one site from other remaining 5 sites (D5, D6,A4,R11,R12). The average enrichment scores for 1%, 2% and 5% of the database compounds was 12.6, 8.96 and 4.42 respectively which was very low compared to the other pharmacophores I and III. Removing these two sites we found the pharmacophore resembled the typical class I type pharmacophore [57]. Our results also showed that most of the active compounds in our dataset belong to this pharmacophore type with fitness more than two. Other than that we also calculated the BEDROC score, RIE, Enrichment scores and AUC using the R package enrichVS (http://cran.r-project.org/web/packages/enrichvs/index.html).

### E pharmacophore validation

In a VS process when a model gets developed its need to be also validated. For the three pharmacophores 1000 decoys and 35 active molecules were selected to check out that whether pharmacophores can find actives and rank them early. Phase findmatches was applied for each set of pharmacophores. For E- Pharmacohore I, II and III a minimum of 4 features out of 5 were given for search in the database. The enrichments calculated by the three pharmacophores are given in Table 1. It was found that for pharmacophore I the enrichments and the BEDROC value was quite low for other two pharmacophores. Also the yield of actives and the goodness of hit were comparatively low as given in Table 2. It was observed for pharmacophore II and III that enrichments scores were quite equal along with percentage yield of actives, specificity and goodness of hit was quite high which indicated that the pharmacophore was able to select active proportion of compounds from the database. We had derived pharmacophore III from II by removing the features D8 and R13 from pharmacophore II containing one donor, one ring aromatic. Table 3 gives the metrics for the donor D8, ring R13 and any one site from the 5 points. It showed that both D8 and R13 sites had a very low score of retrieving ranked actives, low % of yield of actives along with GH score.

**Table 1:** The table shows the enrichment factors, BEDROC value and RIE of the different methods applied in virtual screening

**Method**	**EF(1%)**	**EF(2%)**	**EF(5%)**	**EF(10%)**	**BEDROC(α=20)**	**RIE**
E-pharmacophore I(5 sites)	12	11	10.51	6.8	0.538	7.81
E-pharmacophore II(7 sites)	30	30	13	6.8	0.729	10.6
E-pharmacophore III(5 sites)	30	27	13	6.5	0.706	10.26
ROCS	30	27	13.14	7.42	0.749	10.89
Glide XP	27	21	11.42	6.28	0.629	9.14
Sum score	30	29	**14**.**85**	7.42	**0**.**785**	**11**.**42**
Sum rank	30	24	12	7.42	0.703	10.21
Reciprocal rank	30	30	**17**.**14**	8.85	**0**.**875**	**12**.**73**

**Table 2:** Shows the % yield of actives (Ya), %actives, sensitivity, specificity and GH score of pharmacophores

**Pharmacophore**	**%Yield Of Actives**	**%Actives**	**Sensitivity**	**Specificity**	**Goodness of Hit(GH SCORE)**
E- pharmacophore I	7	68	0.68	0.71	0.157975
E- pharmacophore II	6	74.2	0.742	0.596	0.137378
E- pharmacophore III	**14**.**3**	65.7	0.657	**0**.**86**	**0**.**23349**

**Table 3:** Shows donor D8 and Ring R13 along with any other pharmcophoric point from 5 sites

**Pharmacophore points**	**BEDROC(α=20)**	**RIE**	**%Yield Of Actives**	**%Actives**	**Specificity**	**Goodness of Hit(GH Score)**
D8,R12,R13	0.331	4.81	7.3	51.4	0.774	0.1418355
D8,R13,R11	0.306	4.45	11.11	31.4	0.912	0.1475844
D8,R13,A4	0.183	2.67	5.8	14.2	0.919	0.072601
D8,R13,D5	0.32	4.74	11.8	31.4	0.918	0.153306
D8,D6,R13	0.18	2.72	23.8	14.2	0.984	0.210576

This indicates that D8 and R13 cannot be selected as a pharmacophoric point.

**Figure 4:**
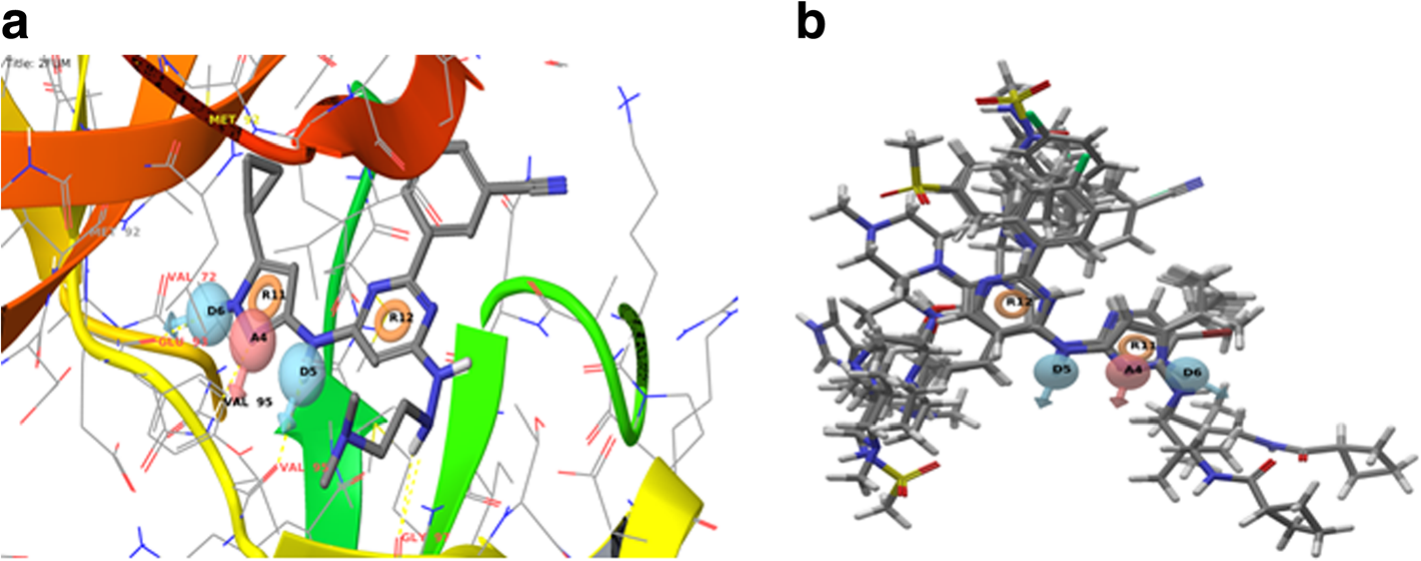
**a) shows the compound VII docked pose at the binding site with e-pharmacophore sites. b**) shows the actives compounds mapped to the e-pharmacophore.

The Table 3 indicates that the two point’s donor D8 and ring R13 cannot be selected as pharmacophoric point for further VS. This was the basis for our selection of pharmacophore III. Pharmacophore I retrieved hits well above 0.1 μM but it was observed that when an extra donor D6 is present in the pharmacophore for example in pharmacophore III activity values decreased sharply below 0.1μM. Pharmacophore I had a very low BEDROC, RIE and enrichment for 1%, 2% and 5% of database hits other than two pharmacophores which means that this pharmacohore was unable to retrieve satisfactory highly ranked hits compared to other two pharmacophores. Figure 4a shows the pharmacophoric sites and the docking pose of compound **VIII** at the active site of *Mycobacterium tuberculosis* PknB and Figure 4b shows the active compounds mapped to e-pharmacophore III.

### Shape based screening

For shape based screening we used the same 1035 dataset used for pharmacophore validation i.e. 1000 decoys and 35 active molecules. For query the docked pose of compound **VIII** was taken. After screening the maximum value of the Tanimoto Combo score attained was 1.418. The roc area obtained by vROCS program was 0.89 which was higher than the pharmacophore search and docking.

### Glide XP docking

**Figure 5:**
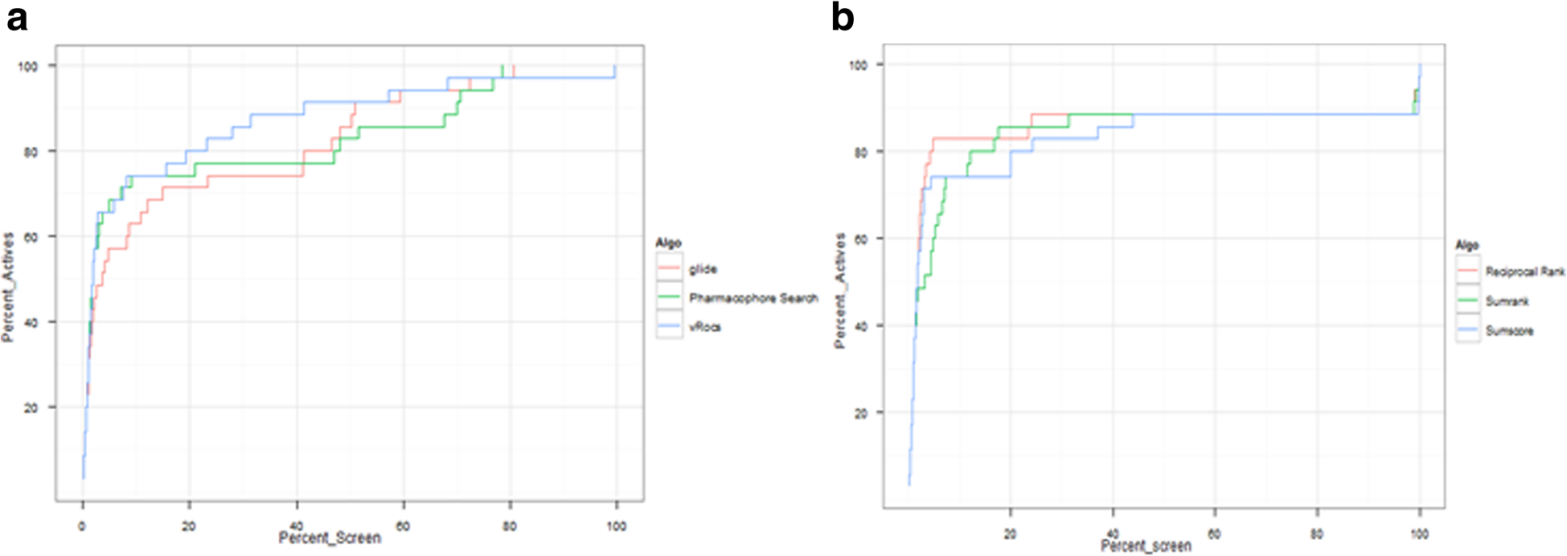
a) It shows the enrichment plots for VS methods and b) shows the enrichment plots for data fusion.

To check the enrichment of the structure based approaches such as docking, we also tested the compounds with Glide XP mode of docking. It approximately took around 72 hrs. to dock 1035 compounds. The roc area calculated for docking is 0.84. Figure 5a gives the enrichment plot of e- pharmacophore III, ROCS and Glide XP docking for all the 1035 database molecules.

### Data fusion

Several data fusion algorithms have been successfully implemented and it has been found that the algorithms scores and ranks compound much better than a single VS method. In this work we have implemented three very simple fusion methods namely sum score, sum rank and reciprocal rank. The study and statistics showed that reciprocal rank performed better among all the other methods with the highest BEDROC score and RIE score followed by Sum score. Both of these algorithms were able to retrieve highly ranked hits. Table 4 shows the area under the roc curve values for VS methods and data fusion. It shows reciprocal rank can retrieve hits much better in 1%, 2%, 5% and 100% of the database.

**Table 4:** **It shows the ROC AUC**’**s of VS methods and data fusion**

**Methods**	**AUC(1%)**	**AUC(2%)**	**AUC(5%)**	**AUC(100%)**
E-pharmacophore III	0.56	0.602	0.649	0.832
ROCS	0.58	0.62	0.62	0.89
Glide XP	0.39	0.44	0.51	0.84
Sum score	**0**.**64**	**0**.**6780**	**0**.**717**	**0**.**90**
Sum rank	0.47	0.49	0.565	0.91
Reciprocal rank	**0**.**72**	**0**.**75**	**0**.**81**	**0**.**96**

### Virtual screening of Asinex database

With e-pharmacophore III giving the best possible BEDROC and RIE score among the other pharmacophore models, we selected this pharmacophore model for our prepared virtual database of Asinex. We selected top 5000 hits for our work in which only 222 hits scored above 2.0 other than that it was very interesting among all the 222 hits, there were only 14 compounds which met all the necessary five sites of the pharmacophore. Most of the compounds in top 222 lacked the D6 donor site of pharmacophore III. But in the active set we found that there were some compounds which lacked this site showed a good docking score with one acceptor and one donor. For shape based screening of 5000 compounds a maximum of 1000 conformers per molecule were generated using the parameters set by Bostrom et al. [42].Then we ran vROCS using the Glide XP docked query for the generated dataset. For vROCS query the maximum Tanimoto combo score attained after screening was 1.19. Ranking of the vROCS results was done by the program itself. Due to time and power limitations we used Glide SP for docking purpose. All the 5000 molecules were ranked in ascending order of the docking score. For data fusion we used reciprocal rank algorithm to rank the compounds as because it performed the best among all the other fusion algorithms. After ranking was done top 10% (500 compounds) of the database hits ranked by reciprocal rank were docked in the 2FUM ATP binding site with Glide XP docking algorithm. Then each of the 500 poses were visually inspected and mapped to the pharmacophoric sites. We selected a list of 45 inhibitors which matched the above limitations and also resembling the pose of compound **VIII**. Additional file 3 contains the structures of 45 compounds.

### Physicochemical space of the PknB inhibitors

**Figure 6:**
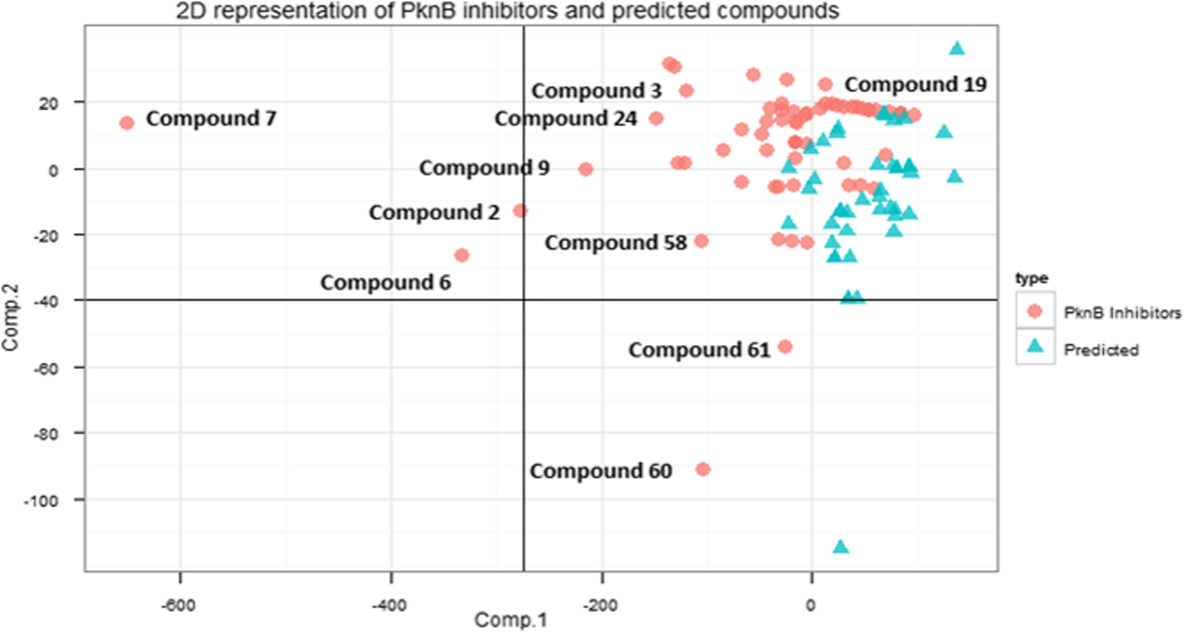
**It shows the Principal component analysis study of the PknB inhibitors and our predicted compounds.** The numbers of the compounds assigned as the positions in the sdf format of Additional file 3.

**Figure 7:**
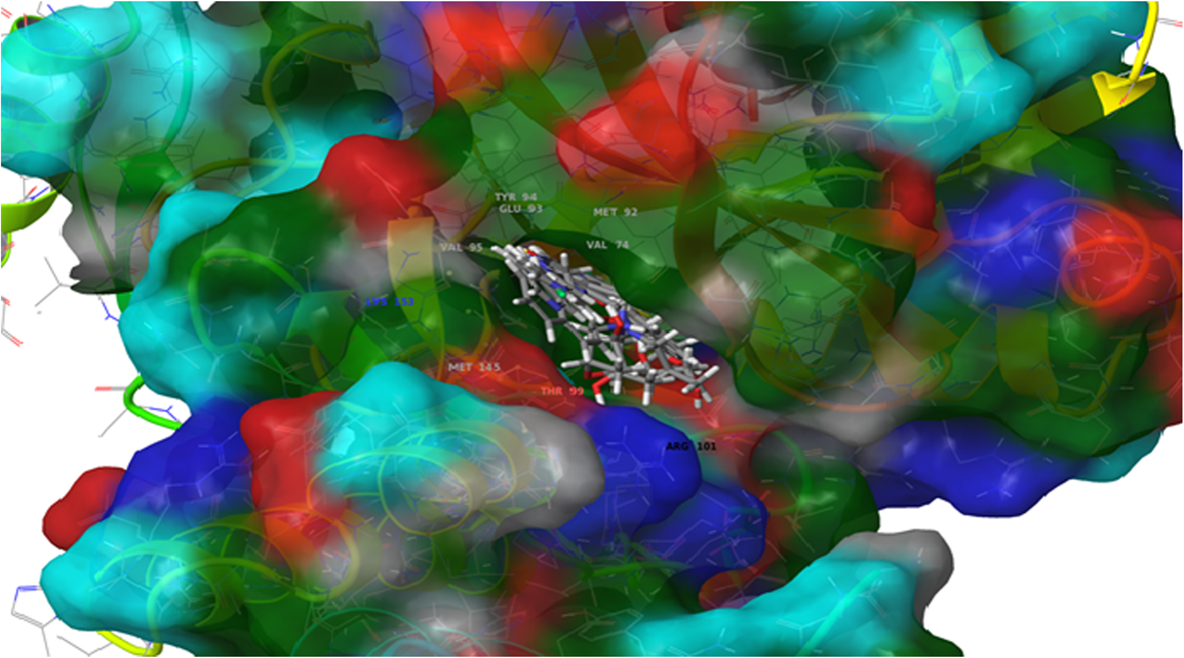
Showing some of the top ranked selected hits binding to the PknB active site.

It is well known that the antibacterial drugs covers a wide range of chemical space [58]. Antibacterials does not follow Lipinski’s rules as they have high polar surface area, low lipophilicity and high molecular weights[57,59]. We studied 12 different physicochemical properties including molecular refractivity, atom polarizabilities, bond polarizabilities, hydrogen bond donors and acceptors, petitjean number, topological polar surface area, number of rotatable bonds, liphophilicity XLogP, molecular weight, topological shape and geometrical shape. The twelve properties were calculated using Chemistry development kit tool developed by Guha (http://rguha.net/code/java/cdkdesc.html) [60]. A principal component analysis (PCA) was done using the 12 chemical properties which transform a set of correlated variables into a number of uncorrelated variables called principal components was done using R princomp function. Principal component 1 and 2 is plotted in the x and y axis which has the maximum variance is shown in Figure 6. The x and y axes are linear combinations of the 12 properties and each data point in the two dimensional graph corresponds to one compound. It has been found that the PknB inhibitors have a wide distribution of in chemical space. It was found that many different set of compounds which were structurally different appeared in different wide positions in chemical space. Some of the compounds are labeled in Figure 6. Our predicted 58 group of compounds fall into the section of the active set of PknB inhibitors which means that these compounds share similar physicochemical properties with the PknB inhibitors. A set of some predicted compounds binding to the active ATP site of PknB is given in Figure 7.

## Conclusion

In this work we have created the *Mycobacterium tuberculosis* PknB pharmacophore model which is can be used for further development high affinity compounds. We have developed three pharmacophore models using e-pharmacophore and came to find out that most of the active compounds in the dataset of 62 compounds resemble the kinase type I pharmacophore [54] which is represented by e-pharmacophore III. Data fusion methods previously being implemented in 2D screening protocols [27] and also now being widely accepted in 3D screening methods. In our work we have used sum score, sum rank and reciprocal rank algorithms. Reciprocal rank algorithm is used in the information retrieval systems in meta search engines [47]. It has been found that previously no one has implemented the reciprocal rank algorithm for data fusion using 3D methods. We have found that reciprocal rank algorithm performed better than sum score and sum rank fusion methods results which indicates that it can rank molecules better in a VS run. After running a virtual screening run we have found identified compounds based on reciprocal rank algorithm and further docking by glide XP and pharmacophore mapping. We did found around 45 compounds which were having one acceptor and one donor and one ring. We also mapped the compounds to the physicochemical space of the PknB inhibitors and found that many of the compounds fall in the same physicochemical region as of PknB inhibitors. The set of 45 compounds in the Additional file 3 could be further processed for experimental validation against PknB.

## Experimental

### Datasets

The following are the datasets used for these experiments.

Additional file 1: The PknB dataset of 62 inhibitors which contains Pubmed ID and IC_50_ values of inhibitors.

Additional file 2: The validation dataset of 1035 compounds in which 35 are active compounds and 1000 are decoys.

Additional file 3: It contains 45 compounds which are visually mapped with pharmacophore and Glide XP docking. It also contains the reciprocal rank scores of the compounds along with the Glide XP docking scores, Tanimoto Combo Score and pharmacophore fitness values.

## Competing interests

The authors have no competing interests in this paper.

## Authors’ contributions

We would like to mention that Mr. AS and Dr. PY have designed the experiment protocol. AS performed the experiments. PY being the corresponding author. OSDDC was involved in regular discussions and supported the work. Dr. DS, Dr. DJW, OSDD Consortium are the co-authors of this paper. Dr. DJW & Dr. DS helped in editing the manuscript. All authors read and approved the final manuscript.

## Supplementary Material

Additional file 1 The PknB dataset of 62 inhibitors which contains Pubmed ID and IC_50_ values of inhibitors. Click here for file

Additional file 2 The validation dataset of 1035 compounds in which 35 are active compounds and 1000 are decoys. Click here for file

Additional file 3 **It contains 45 compounds which are visually mapped with pharmacophore and Glide XP docking.** It also contains the reciprocal rank scores of the compounds along with the Glide XP docking scores, Tanimoto Combo Score and pharmacophore fitness values. Click here for file
